# Comparative evaluation of supplemental zilpaterol hydrochloride sources on growth performance, dietary energetics and carcass characteristics of finishing lambs

**DOI:** 10.5713/ajas.18.0152

**Published:** 2018-12-21

**Authors:** A. Rivera-Villegas, A. Estrada-Angulo, B. I. Castro-Pérez, J. D. Urías-Estrada, F. G. Ríos-Rincón, D. Rodríguez-Cordero, A. Barreras, A. Plascencia, V. M. González-Vizcarra, J. F. Sosa-Gordillo, R. A. Zinn

**Affiliations:** 1 Facultad de Medicina Veterinaria y Zootecnia, Universidad Autónoma de Sinaloa, Culiacán 80246, Sinaloa, México; 2 Instituto de Investigaciones en Ciencias Veterinarias, Universidad Autónoma de Baja California, Mexicali 21386, Baja California, México; 3 Department of Animal Science, University of California, Davis, CA 95616, USA

**Keywords:** Finishing Lambs, Zilpaterol Hydrochloride, Generics, Dietary Energy, Carcass, Visceral Mass

## Abstract

**Objective:**

We compare the effects of three different approved sources of supplemental zilpaterol on growth-performance responses and carcass characteristics of finishing lambs.

**Methods:**

Twenty four Pelibuey×Katahdin lambs (46.75±2.43 kg) were used in a 33-day feeding trial. Lambs were fed a dry rolled corn-based finishing diet. Treatments consisted of the non-supplemental basal diet (Control) versus the basal diet supplemented with 125 mg zilpaterol/kg of diet (as fed basis) from three commercial sources marketed in Mexico: Zilmax (ZIL), Grofactor, and Zipamix.

**Results:**

Compared to controls, zilpaterol (ZH) supplementation did not affect dry matter intake (DMI), but increased carcass adjusted daily weight gain (ADG, 36.7%), gain efficiency (34.2%), and dietary net energy (26.0%), and decreased (23.4%) the ratio of observed:expected DMI. Compared to controls, supplemental ZH increased hot carcass weight (6.4%), dressing percentage (3.2%), *m. longissimus thoracis* (LM) area (15.6%), and shoulder muscle:fat ratio (28.7%), but decreased kidney-pelvic-heart fat, and fat thickness. Supplemental ZH increased 10.9% and 14.3% whole cut weight of loin and leg, respectively, and the proportion (as percentage of cold carcass weight) of leg (4.3%). These increases were reflected in greater forequarter and hindquarter weights. Lambs fed ZH increased (4.6%) empty body weight (EBW) and reduced (14.7%) liver/spleen weight (as g/kg EBW). Likewise, ZH supplementation tended (p = 0.08) to lower (8.9%) visceral fat. Growth performance, energetic efficiency, hot carcass weight, dressing percentage, LM area and whole cuts were not different across supplemental ZH sources. However, compared with non-supplemented controls, only ZIL appreciably decreased carcass fat distribution, including fat thickness, percentage kidney pelvic and heart fat, shoulder fat, and visceral fat.

**Conclusion:**

Supplemental ZH increases ADG, gain efficiency, carcass dressing percentage, and LM area. The magnitude of these responses was similar among ZH sources. Nevertheless, compared with non-supplemented controls, only ZIL appreciably decreases carcass fat. The basis for this is uncertain, but indicative that some practical differences in zilpaterol bio-equivalency may exist across commercial sources tested.

## INTRODUCTION

Zilpaterol hydrochloride (ZH), a beta adrenergic agonist, is an FDA-approved feed additive for beef cattle [1]. It was originally patented and marketed under the trade name Zilmax (ZIL; MSD, Summit, NJ, USA). However, following patent expiration outside the USA, additional “generic” forms of the compound have been approved for marketing in countries where the use of ZH as a feed additive is authorized. Notwithstanding potentially lower product costs, industry acceptance of “generic” forms of feed additives is limited [2]. The basis for this include: perceived differences in quality control during manufacturing and marketing; uniformity; purity; drug particle size and carrier, and their associated effects on product distribution during feed mixing and delivery; manufacturer product support; and demonstrated bio-equivalency. Supplementing finishing lambs with ZH at the rate of 6 mg/kg diet dry matter (DM) increase average daily gain (ADG), gain efficiency [3–5], carcass weight and dressing percentage [5,6]. These effects help to increase gain efficiency, particularly during the late finishing phase when cost of gain is greatest. The objective of the present study was to compare the effects of three approved sources of ZH (MEX SAGARPA, 2016; registration Q-0042-401, Q-7833-242, and Q-0273-205) marketed under the trademark Grofactor (GRO; Laboratorios Virbac México, Guadalajara, Mexico), Zipamix (ZIPA; Pisa Agropecuaria, Guadalajara, Mexico), and ZIL (MSD, Salud Animal Mexico, Estado de Mexico, Mexico) on growth performance, dietary energetics and carcass characteristics of finishing lambs.

## MATERIALS AND METHODS

### Diets, animals and experimental design

This experiment was conducted at the Universidad Autónoma of Sinaloa Feedlot Lamb Research Unit, located in the Culiacán, México (24° 46′ 13″ N and 107° 21′ 14″ W). Culiacán is about 55 m above sea level, and has a tropical climate. Average daily minimum and maximum air temperature during the trial was 25.9°C and 33.9°C (average = 29.9°C), respectively. Average daily relative humidity was 78.8%. All animal management procedures were conducted within the guidelines of locally-approved techniques for animal use and care: NOM-051-ZOO-1995: Humanitarian care of animals during mobilization of animals; NOM-062-ZOO-1995: Technical specifications for the care and use of laboratory animals. Livestock farms, farms, centers of production, reproduction and breeding, zoos and exhibition halls, must meet the basic principles of animal welfare; NOM-024-ZOO-1995: Animal health stipulations and characteristics during transportation of animals, and NOM-033-ZOO-1995: Humanitarian care and animal protection during slaughter process.

Twenty-four Pelibuey×Katahdin (46.7±2.4 kg initial shrunk weight) crossbred intact male lambs were used in a 33-d growth-performance experiment to evaluate the treatment effects on growth performance, dietary energetics, carcass traits, and visceral organ mass. Prior to initiation of the study, lambs were treated for endoparasites (Albendaphorte 10%, Animal Health and Welfare, México City, México), and injected with 1×10^6^ IU vitamin A (Synt-ADE, Fort Dodge, Animal Health, Mexico City, México). Lambs then adapted to the control diet (Table 1) for a period of 7 weeks. Upon initiation of the experiment, lambs were individually weighed (electronic scale; TORREY TIL/S: 107 2691, TOR REY electronics Inc, Houston TX, USA), grouped by weight into six uniform weight blocks, and assigned to pens (1 lamb/pen). Individual pens were 6 m^2^ with overhead shade, automatic waterers and 1 m fence-line feed bunks. Dietary treatments (Table 1) consisted of a corn-based finishing diet supplemented with no zilpaterol (Control), or the, same basal diet plus the label dosage (125 mg of product/kg diet, as-fed basis) as ZIL (MSD Salud Animal Mexico, Santiago Tianguistenco, Mexico), GRO (Laboratorios Virbac México, Mexico), or ZIPA (Pisa Agropecuaria, Mexico). According to the label, all products tested contained 4.8% ZH. Thus, the dosage of 125 mg of product/kg diet corresponds to a dietary ZH concentration of 6 mg/kg (as feed basis). Supplemental ZH was hand-weighed using a precision balance (Ohaus, mod AS612, Pine Brook, NJ, USA), and premixed for 5 min with the other minor dietary ingredients (urea, limestone, and trace mineral salt) before incorporation into complete mixed basal diet using a 2.5 m^3^ capacity paddle mixer (mod 30910-7, Coyoacán, México). To avoid contamination, the mixer was thoroughly cleaned between each treatment. The ZH was supplemented for 30 d followed by a 3-d preharvest withdrawal when all lambs received the non-supplemented basal control diet. The four dietary treatments were randomly assigned to pens within each weight block in a randomized complete block design. Lambs were allowed *ad libitum* access to dietary treatments. Fresh feed was provided twice daily at 0800 and 1400 h in a 40:60 proportion (as feed basis). Daily feed allotments to each pen were adjusted to allow for approximately 5% residual feed remaining in feed bunk at time of the morning feeding. Feed bunks were visually assessed between 0740 and 0750 h each morning, residual feed was collected and weighed for determination of daily feed intake. Adjustments, in daily feed delivery were provided at the afternoon feeding. Lambs were individually weighed at the beginning of the trial and at harvest. The initial shrunk body weight (SBW) was determined as full body weight×0.96 (adjustment for gastrointestinal fill). Upon completion of the study, all lambs were weighed following an 18 h fast (feed but not drinking water was withdrawn) to obtain final SBW. Final SBW was adjusted for hot carcass weight (HCW) by dividing the individual HCW by the average dressing percentage (0.6120) for all lambs.

### Sample analysis

Dietary treatments were subjected to the following analyses [7]: DM (oven drying at 105°C until no further weight loss; method 930.15); crude protein (N×6.25, method 984.13); ash (method 942.05), and ether extract (method 920.39). The neutral detergent fiber (NDF) fraction was determined according to Van Soest et al [8] [corrected for NDF-ash, incorporating heat stable α-amylase (Ankom Technology, Macedon, NY, USA) at 1 mL per 100 mL of NDF solution (Midland Scientific, Omaha, NE, USA)]. Feed and orts were sampled daily for DM analysis (oven-drying at 105°C until constant weight, method 930.15) [7]. The ZH concentrations for the various sources (blind samples) were assayed by MSD Quality Control Laboratory (MSD Salud Animal Mexico, Mexico).

### Calculations

Average daily gain was determined as the difference in initial SBW and carcass adjusted final SBW divided by 33 (days on test). Gain efficiency was determined as the ADG divided by corresponding dry matter intake (DMI). The estimation of expected DMI was performed based on observed ADG and average SBW according to the following equation: expected DMI, kg/d = (EM/NE_m_)+(EG/NE_g_), where EM is the energy required for maintenance, Mcal/d (0.056×SBW^0.75^) [9], EG is the energy gain, Mcal/d (0.276×ADG×SBW^0.75^) [9], and net energy for maintenance (NE_m_) and net energy for gain (NE_g_) of the diet are 2.11 and 1.44 Mcal/kg, respectively (derived from tabular values based on the ingredient composition of the diet [10]. The coefficient (0.276) is based on a mature weight of 113 kg for Pelibuey×Kathdin male lambs [3]. Dietary NE was estimated by means of the quadratic formula: $x=(-\text{b}-\sqrt{b^{2}-4ac})/2\text{c}$, where *x* = NE_m_, *a* = −0.41EM, *b* = 0.877EM+ 0.41DMI+EG, and *c* = −0.877 DMI in accordance to Zinn et al [11].

### Carcass and visceral mass data

All lambs were harvested on the same day. After humanitarian sacrifice, lambs were skinned, and the gastrointestinal organs were separated and weighed. After carcasses (with kidneys and internal fat included) were chilled in a cooler at −2°C to 1°C for 48 h, the following measurements were obtained: i) fat thickness perpendicular to the *m. longissimus thoracis* (LM), measured over the center of the ribeye between the 12th and 13th rib; ii) LM surface area, measure using a grid reading of the cross-sectional area of the ribeye between 12th and 13th rib, and iii) kidney, pelvic and heart fat (KPH). The KPH was removed manually from the carcass, and then weighed and reported as a percentage of the cold carcass weight [12]. Each carcass was split into two halves. The left side was fabricated into wholesale cuts, without trimming, according to the North American Meat Processors Association guidelines [13]. Rack, breast, shoulder and foreshank were obtained from the foresaddle, and the loins, flank and leg from the hindsaddle. The weights of each cut were subsequently recorded. The tissue composition of shoulder was assessed using physical dissection by the procedure described by Luaces et al [14].

All tissue weights were reported on a fresh tissue basis. Organ mass was expressed as g/kg final empty body weight (EBW). Final EBW represents the final SBW minus the total digesta weight. Full visceral mass was calculated by the summation of all visceral components (stomach complex+small intestine+large intestine+liver+lungs+heart), including digesta. The stomach complex was calculated as the digesta-free sum of the weights of the rumen, reticulum, omasum and abomasum. The weights of the heart and lungs, and the weights of liver and spleen were recorded together.

### Statistical analysis

Growth performance (weight gain, feed intake, gain efficiency), dietary energetics, carcass data and visceral mass data were analyzed as a randomized complete block design using the MIXED procedure of SAS [15], where initial weight was the blocking criterion (blocks = 6), and lamb was considered as the experimental unit. The treatment means were separated using the least significant difference test (Tukey’s Test). Treatment effects were considered significant when the value of p ≤ 0.05, and were identified as trends when the value of p>0.05 and ≤0.10.

## RESULTS

Assayed ZH concentrations were within the expected range (43.2 to 53.8 g/kg), averaging 47.8, 47.3, and 51.2 g ZH/kg of product for ZIL, GRO, and ZIPA, respectively. Thus, based on average as-fed intake, ZH intake averaged 7.92, 7.73, and 8.1 mg/d, corresponding to 0.157, 0.153, and 0.162 mg ZH/kg SBW for lambs fed ZIL, GRO, and ZIPA, respectively.

Treatment effects on growth performance and dietary energetics are shown in Table 2. Compared to controls, ZH supplementation did not affect (p>0.10) DMI, but increased (p<0.05) carcass adjusted ADG (36.7%), gain efficiency (34.2%) and dietary NE (26.0%). Accordingly, ZH supplementation markedly decreased (23.4%, p<0.05) the ratio of observed: expected DMI. At comparable levels of ZH intake, growth performance and energetic efficiency responses of feedlot lambs were not different (p>0.10) across supplemental ZH sources.

Treatment effects on carcass composition are shown in Table 3. Compared to controls, supplemental ZH increased (p<0.05) HCW (6.4%), dressing percentage (3.2%), and LM area (15.6%). Difference in HCW, dressing percentage and LM area among ZH sources was not appreciable (p>0.10). Compared to control, ZIL supplemented lambs had less (p<0.05) KPH and fat thickness (21.7% and 13.2%, respectively) and greater (28.7%, p<0.05) shoulder muscle:fat ratio.

The ZH supplementation increased (p≤0.02) 10.9 and 14.3% whole cut weight of loin and leg, respectively, and increased 4.3% the proportion of leg (as percentage of cold carcass weight). These increases were reflected in greater forequarter (p = 0.03) and hindquarter (p<0.01) weights. Differences among ZH sources on whole cuts were not appreciable (p> 0.10; Table 4).

Treatment effects on visceral mass are shown in Table 5. Compared to controls, ZH supplementation increased (4.6%, p<0.01) EBW and reduced (14.7%, p<0.01) liver/spleen weight (as g/kg EBW). Likewise, ZH supplementation tended (p = 0.08) to lower (8.9%) visceral fat. However, supplemental ZH did not affect heart/lung weight (p = 0.32), stomach complex (p = 0.29), or empty weight of intestines (p = 0.67). Lambs fed ZIL or ZIPA had less (15.5%, p<0.05) visceral fat as a proportion of EBW than control or GRO supplemented lambs.

## DISCUSSION

Optimal daily ZH dosage for feedlot cattle is between 0.15 to 0.165 mg/kg body weight (BW) [16,17]. In practice, this corresponds to a dietary concentration of 6.7 mg/kg diet DM (6 mg/kg air-dry feed, 90% DM basis). Zilpaterol is not currently labeled as a feed additive for lambs. The numerous studies conducted thus far in feedlot lambs adopted the labeled dosage as indicated for feedlot cattle [3,5,6,18].

Enhancements in ADG, gain efficiency and dietary energetics when lambs were supplemented with ZH are consistent with previous reports [3,19]. In previous studies, ZH supplementation of finishing diets for feedlot lambs increased ADG by 20.1% to 40.6% and gain efficiency by 16.5% to 43.3%. The observed enhancements in ADG and gain efficiency in the present study fall within those ranges. However, the decrease (13.3% to 16.2%) in observed:expected DMI ratio due to ZH supplementation (23.4%) was greater than previously observed [3,5,19,20].

Increased HCW, LM area, and dressing percentage, and reduced backfat thickness with ZH supplementation has been a consistent response in feedlot lambs [18,21]. Increased LM area is partially explained by the greater ADG [22], whereas the increased dressing percentage is expected due to greater carcass protein accretion with no change in digestive tract fill [6,23]. The increased LM area is also consistent with increased shoulder muscle [21].

Increased carcass cutability due to ZH supplementation has been consistent response in feedlot cattle [16], with the more pronounced effect occurring in the hindquater [17]. However, in lambs, the effects of ZH supplementation on carcass cutability is less consistent. Whereas in some studies [4,24] supplemental ZH increased loin and leg (as observed in the present experiment), in others [25,26] supplemental ZH did not affect lamb carcass wholesale cuts. The basis for this is uncertain. Estrada-Angulo et al [3] observed that factors such as diet energy density, age, genetics, and ZH dosage level may influence response to supplemental ZH. Comparative effects of different ZH sources on carcass cutability has not been previously reported. However, because the dietary concentration of ZH was similar for each of the three sources, absence of effects of ZH source on carcass cutability is expected.

The effects of β-agonist on non-carcass organs has received limited attention. The β-agonist salbutamol decreased liver mass in pigs [27]. Likewise, supplemental ZH decreased liver mass in feedlot cattle [28]. Decreased liver mass has also been a consistent observation in feedlot lambs receiving ZH [5,23].

Effects of ZH on visceral organs has been attributed to the differences in the abundance of β-agonist receptors subtypes in these tissues [17]. In as much as an appreciable proportion of energy expenditure can be attributed to maintenance of visceral organs, especially the liver and gastrointestinal tract [29], reductions in visceral organ mass could contribute to the increased energy efficiency observed when dietary β-agonists are fed.

Increased carcass dressing percentage explained 66% (65 g/d) of the increase (98 g/d) in carcass adjusted ADG in ZH supplemented lambs. Enhanced daily gain accounts for 55% of the net economic value of ZH supplementation (benefit to the feeder), while increased carcass yield accounts for 45% of the net value (benefit to the meat packer and retailer). Thus, the economic benefit to ZH supplementation is optimized through integrated production and meat purveying systems (Table 6).

Avendaño-Reyes et al [30] compared GRO vs ZIL in crossbreed bulls (75% *Bos indicus* and remainder *Bos taurus*) in a 34-d finishing trial (30 days of ZH supplementation and 4 days withdrawal). Bulls ingested an average of 0.134 and 0.139 mg ZH/kg BW of GRO and ZIL, respectively. Supplemental ZH enhanced ADG and gain efficiency. However, growth performance responses were not affected by ZH source. Likewise, in the present study differences in growth performance responses in lambs fed different ZH sources were not appreciable.

Avendaño-Reyes et al [30] did not detect an effect of supplemental ZH source on carcass characteristics and organ weights of feedlot cattle. Likewise, in the present study differences among ZH sources on carcass characteristics and organ weights were not appreciable. In contrast, ZH sources differed in their effects on fat distribution. Compared with control, supplemental GRO and ZIPA did not affect (p>0.10) fat distribution. Whereas supplemental ZIL decreased (p<0.05) carcass fat thickness, KPH, and shoulder muscle:fat ratio. In as much as all ZH products provided a similar zilpaterol dosage, this outcome was not expected.

## CONCLUSION

Supplemental ZH increases ADG, gain efficiency, carcass dressing percentage and LM area, while reducing observed to expect DMI in feedlot lambs. The magnitude of these responses were similar among ZH sources. Nevertheless, compared with non-supplemented controls, only ZIL appreciably decreases carcass fat. The basis for this is uncertain, but indicative that some practical differences in zilpaterol bio-equivalency may exist across commercial sources tested.

## Figures and Tables

**Table 1:** Composition of basal diet supplemented with different sources of zilpaterol hydrochloride

Item	Zilpaterol sources			

	None	Zilmax	Grofactor	Zipamix
Ingredient composition (%)				
Dry-rolled corn	64.50	64.50	64.50	64.50
Sudan grass hay	12.00	12.00	12.00	12.00
Soybean meal	10.00	10.00	10.00	10.00
Zilpaterol hydrocholride1)				
Zilmax	----	+++	----	----
Grofactor	----	----	+++	----
Zipamix	----	----	----	+++
Molasses cane	8.00	8.00	8.00	8.00
Urea	0.43	0.43	0.43	0.43
Tallow	3.00	3.00	3.00	3.00
Trace mineral salt2)	2.07	2.07	2.07	2.07
DM	87.55	87.55	87.55	87.55
Chemical composition (DM basis)				
Total crude protein (%)	13.23	13.23	13.23	13.23
Ether extract (%)	5.57	5.57	5.57	5.57
NDF (%)	16.65	16.65	16.65	16.65
Calculated net energy3) (Mcal/kg)				
Maintenance	2.11	2.11	2.11	2.11
Gain	1.44	1.44	1.44	1.44

DM, dry matter; NDF, neutral detergent fiber.

1) Sources of zilpaterol hydrochloride (Zilmax [ZIL, MSD Salud Animal Mexico, Estado de Mexico, Mexico]; Grofactor [GRO, Laboratorios Virbac México, Guadalajara, Mexico], and Zipamix [ZIPA, Pisa Agropecuaria, Guadalajara, Mexico]) were supplemented to provide 6 mg zilpaterol hydrochloride/kg diet (as-fed basis).

2) Mineral premix contained: calcium, 28%; phosphorous, 0.55%; magnesium, 0.58%; potassium, 0.65%; NaCl, 15%; vitamin A, 1,100 IU/kg; vitamin E, 11 UI/kg.

3) Based on tabular net energy (NE) values for individual feed ingredients [10].

**Table 2:** Effect of source of dietary zilpaterol hydrochloride on 33-d feedlot growth performance and dietary energy of lambs

Item	Treatments1)				SEM

	None	Zilmax	Grofactor	Zipamix	
Live weight (kg)2)					
Initial	45.37	45.63	45.63	45.68	0.279
Final	50.93^a^	54.55^b^	54.20^b^	54.50^b^	0.723
Average daily gain (kg)	0.168^a^	0.270^b^	0.260^b^	0.267^b^	0.021
Dry matter intake (kg/d)	1.115	1.160	1.145	1.152	0.044
ADG/DMI	0.151^a^	0.233^b^	0.226^b^	0.230^b^	0.012
Dietary NE (Mcal/kg)					
Maintenance	2.05^a^	2.60^b^	2.56^b^	2.59^b^	0.07
Gain	1.38^a^	1.87^b^	1.83^b^	1.86^b^	0.06
Observed:expected dietary NE ratio					
Maintenance	0.97^a^	1.23^b^	1.21^b^	1.23^b^	0.03
Gain	0.96^a^	1.29^b^	1.27^b^	1.29^b^	0.04
Observed to expected DMI	1.04^a^	0.79^b^	0.81^b^	0.79^b^	0.03

SEM, standard error of the mean; ADG, average daily gain; DMI, dry matter intake; NE, net energy.

1) Sources of zilpaterol hydrochloride (Zilmax [ZIL, MSD Salud Animal Mexico, Estado de Mexico, Mexico]; Grofactor [GRO, Laboratorios Virbac México, Guadalajara, Mexico], and Zipamix [ZIPA, Pisa Agropecuaria, Guadalajara, Mexico]) were supplemented to provide 6 mg zilpaterol hydrochloride/kg diet (as-fed basis).

2) Initial live weights (LW) was reduced 4% to adjust for the gastrointestinal fill; final weight was adjusted for HCW by dividing individual HCW by the average dressing percentage for all lambs (0.6119).

a,b Means a row with different superscripts differ (p<0.05).

**Table 3:** Effect of source of dietary zilpaterol hydrochloride on carcass characteristics

Item	Treatments1)				SEM

	None	Zilmax	Grofactor	Zipamix	
Hot carcass weight (kg)	31.17^a^	33.38^b^	33.25^b^	33.35^b^	0.44
Dressing percentage	59.73^a^	62.16^b^	61.29^ab^	61.61^b^	0.06
Cold carcass weight (kg)	30.50^a^	32.80^b^	32.67^b^	32.73^b^	0.43
LM area (cm^2^)	18.04^a^	20.83^b^	21.60^b^	21.69^b^	0.55
Fat thickness (cm)	0.273^ab^	0.237^c^	0.258^bc^	0.298^a^	0.012
Kidney pelvic and heart fat (%)	3.36^a^	2.63^b^	2.90^ab^	2.86a^b^	0.17
Shoulder composition (%)					
Muscle	63.85	65.87	65.64	65.40	0.65
Fat	16.84^a^	13.97^b^	15.80^ab^	15.68^ab^	0.85
Muscle to fat ratio	3.76^a^	4.84^b^	4.26^ab^	4.23^ab^	0.35

SEM, standard error of the mean; LM, *m. longissimus thoracis*.

1) Sources of zilpaterol hydrochloride (Zilmax [ZIL, MSD Salud Animal Mexico, Estado de Mexico, Mexico]; Grofactor [GRO, Laboratorios Virbac México, Guadalajara, Mexico], and Zipamix [ZIPA, Pisa Agropecuaria, Guadalajara, Mexico]) were supplemented to provide 6 mg zilpaterol hydrochloride/kg diet (as-fed basis).

a,b,c Means a row with different superscripts differ (p<0.05).

**Table 4:** Effect of source of dietary zilpaterol hydrochloride on whole cuts

Item	Treatments1)				SEM

	None	Zilmax	Grofactor	Zipamix	
Whole cuts (kg)					
Forequater	6.57	6.99	7.01	6.99	0.15
Hindquarter	5.60^a^	6.31^b^	6.37^b^	6.25^b^	0.12
Shoulder	2.37	2.42	2.59	2.49	0.07
Shoulder IMPS206	1.40	1.39	1.37	1.43	0.09
Leg IMPS233	3.72^a^	4.23^b^	4.14^b^	4.16^b^	0.09
Loin IMPS231	1.12^a^	1.29^ab^	1.36^b^	1.27^ab^	0.06
Rack IMPS204	1.08	1.10	1.15	1.15	0.04
Short rib	1.12	1.28	1.23	1.21	0.06
Flank IMPS232	0.76	0.79	0.85	0.81	0.04
Breast IMPS209	0.60	0.81	0.68	0.71	0.08
Whole cuts (as percentage of cold carcass weight)					
Forequater	43.07	42.55	42.90	42.69	0.61
Hindquarter	36.77^a^	38.55^b^	39.07^b^	38.12^ab^	0.47
Shoulder	15.52	14.74	15.88	15.19	0.38
Shoulder IMPS206	9.22	8.46	8.31	8.80	0.56
Leg IMPS233	24.46^a^	25.84^b^	25.43^ab^	25.39^ab^	0.43
Loin IMPS231	7.34	7.87	8.33	7.82	0.35
Rack IMPS204	7.05	6.70	7.03	7.08	0.23
Short rib	7.35	7.75	7.52	7.40	0.32
Flank IMPS232	4.97	4.84	5.25	4.91	0.25
Breast IMPS209	3.94	4.90	4.19	4.24	0.47

SEM, standard error of the mean.

1) Sources of zilpaterol hydrochloride (Zilmax [ZIL, MSD Salud Animal Mexico, Estado de Mexico, Mexico]; Grofactor [GRO, Laboratorios Virbac México, Guadalajara, Mexico], and Zipamix [ZIPA, Pisa Agropecuaria, Guadalajara, Mexico]) were supplemented to provide 6 mg zilpaterol hydrochloride/kg diet (as-fed basis).

a,b Means a row with different superscripts differ (p<0.05).

**Table 5:** Effect of source of supplement zilpaterol chlorhydrate on visceral organ mass

Item	Treatments1)				SEM

	None	Zilmax	Grofactor	Zilpamix	
Gastrointestinal tract fill (kg)	4.33	3.48	3.84	4.01	0.29
Empty body weight (kg)	47.87	50.22	50.24	50.11	0.50
Empty body weight (% of full weight)	91.73^a^	93.51^b^	92.85^ab^	92.60^ab^	0.49
Full viscera (kg)	7.65	6.68	7.19	7.31	0.32
Organs (g/kg, empty body weight)					
Stomach complex	28.15	26.21	26.34	25.96	0.79
Intestines	41.33	40.11	40.39	40.57	1.97
Liver/spleen	21.12^a^	17.48^b^	17.64^b^	18.92^b^	0.69
Heart/lungs	20.69	20.28	19.10	19.67	0.84
Visceral fat	33.76^a^	28.37^b^	34.52^a^	29.31^b^	1.41

SEM, standard error of the mean.

1) Sources of zilpaterol hydrochloride [Zilmax (ZIL, MSD Salud Animal Mexico, Estado de Mexico, Mexico); Grofactor (GRO, Laboratorios Virbac México, Guadalajara, Mexico), and Zipamix (ZIPA, Pisa Agropecuaria, Guadalajara, Mexico)] were supplemented to provide 6 mg zilpaterol hydrochloride/kg diet (as-fed basis).

a,b Numbers in the same row with different superscript letters differ (p<0.05).

**Table 6:** Costs and income of different sources of supplemental zilpaterol estimated by animal during the experimental phase (33 d)

Item	Treatments			

	None	Zilmax	Grofactor	Zipamix
Costs				
Feed1)	7.77	8.08	7.98	8.03
Processing practice2)	1.39	1.39	1.39	1.39
Zilpaterol supplementation3)	--	0.604	0.441	0.432
Total cost/phase	9.16	10.07	9.81	9.85
Income (selling LW)4)	12.97	20.81	19.96	20.58
Net income	3.81	10.73	10.15	10.73
Difference	---	+6.92	+6.34	+6.92
Income (selling carcass)5)	14.76	24.64	23.34	24.15
Net income	5.32	14.28	13.25	14.02
Difference	---	+8.96	+7.93	+8.70

1) Price of feed = $211.1 US dollar/t.

2) Include: cattle earring, vitamin and dewormed.

3) Price of zilpaterol sources, US dollar/kg: Zilmax = $138.88; Grofactor = $102.77; Zipamix = $100.00. All zilpaterol sources were incorporated in diet at 125 g/t.

4) Calculated as: (Final weight–initial weight)×2.33. The selling price as LW is $2.33 US dollars.

5) Calculated as: [(Final weight–initial weight)×dressing percentage]×4.44. The selling price as carcass is 4.44 US dollars.
